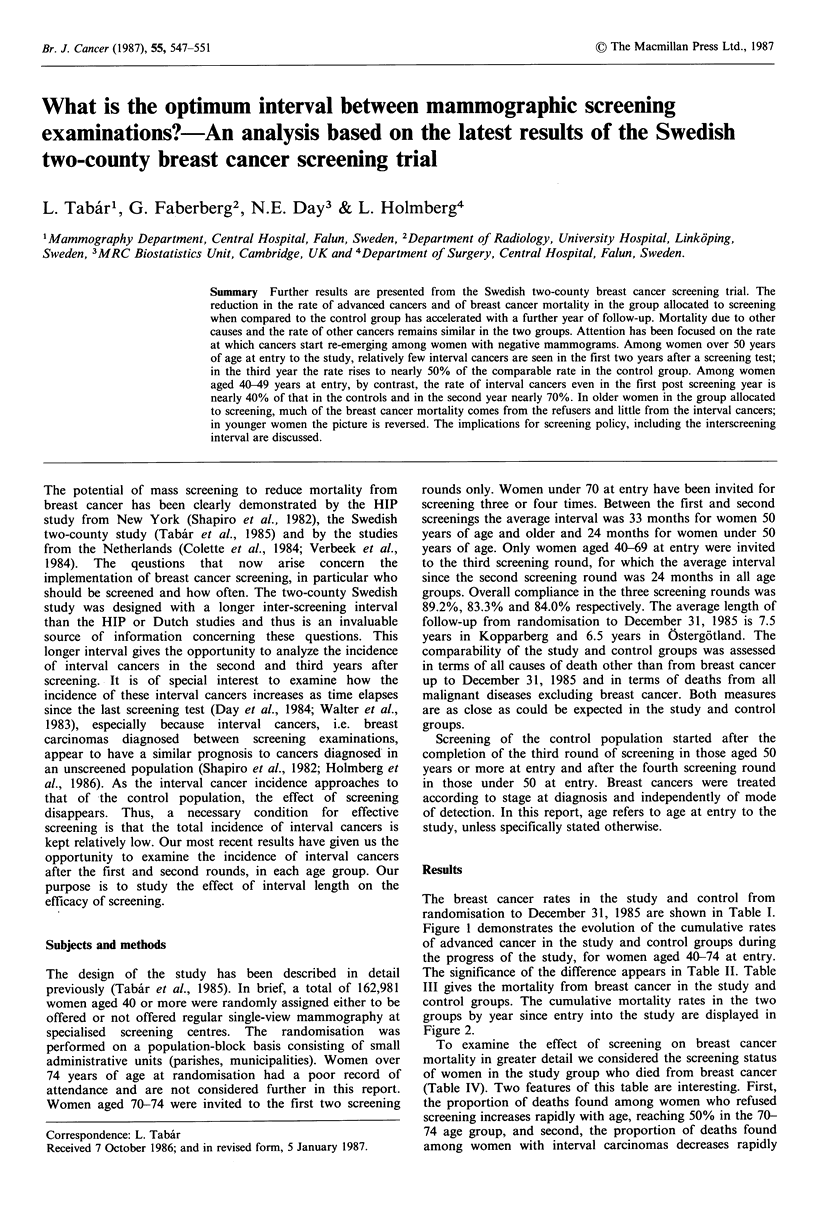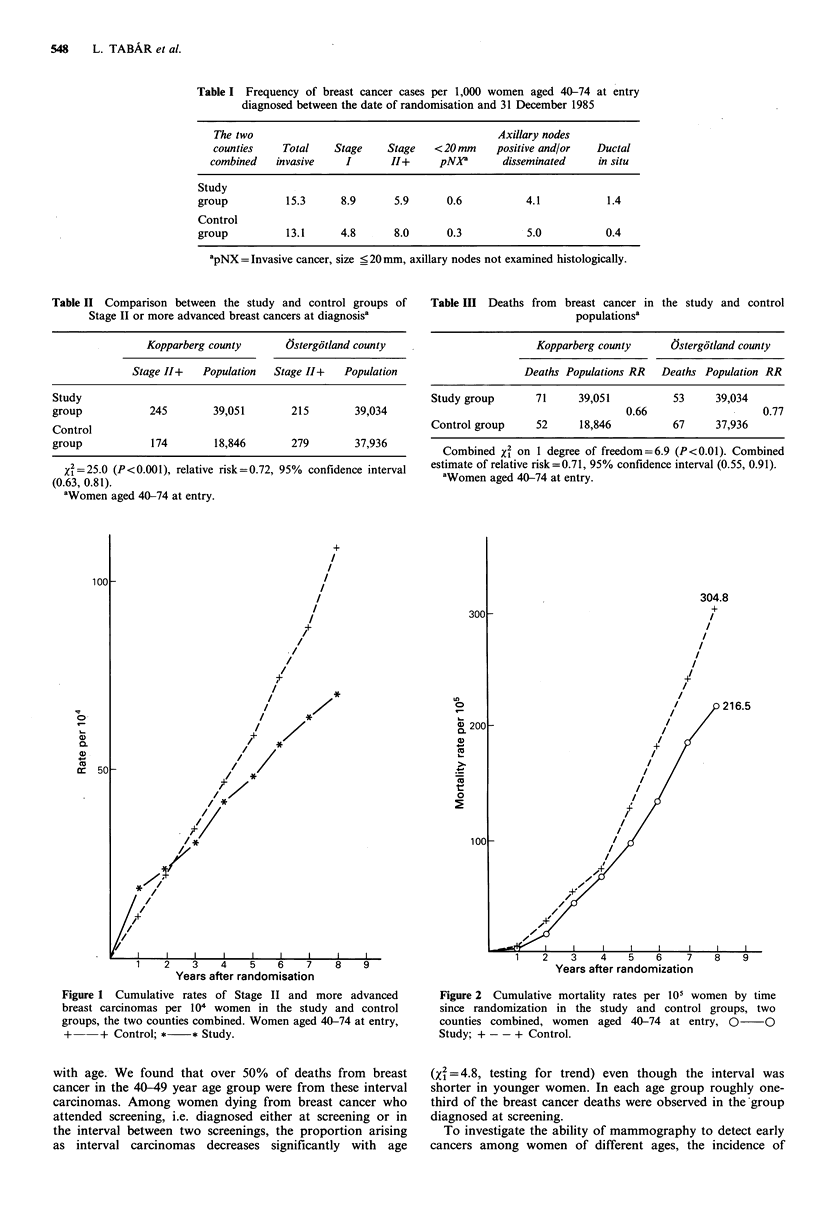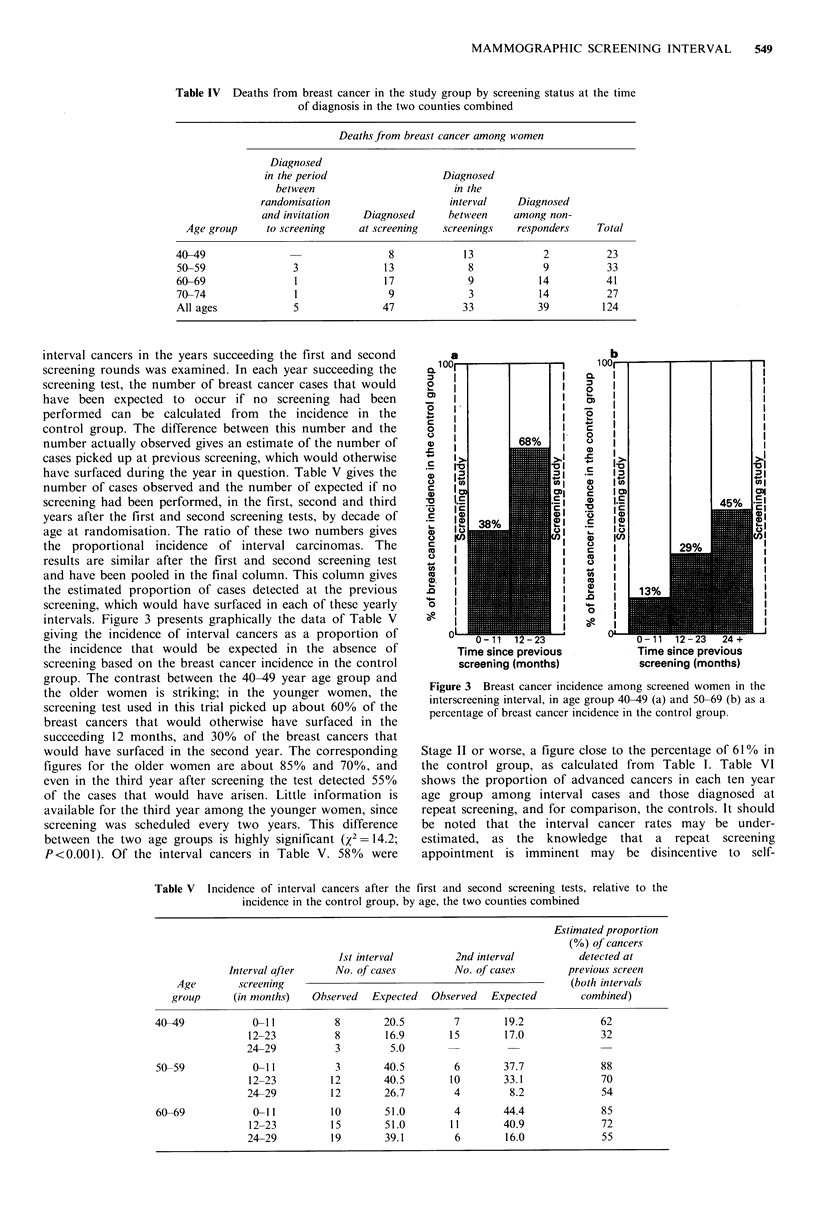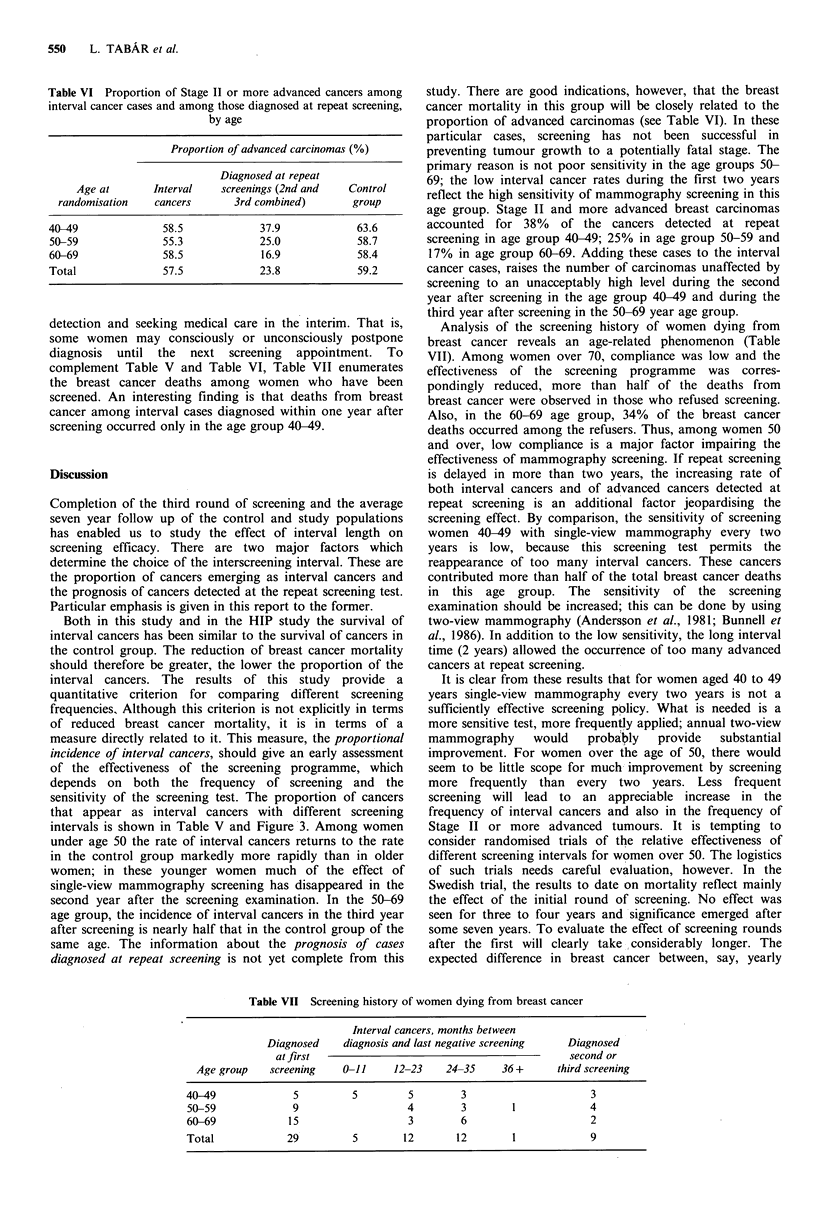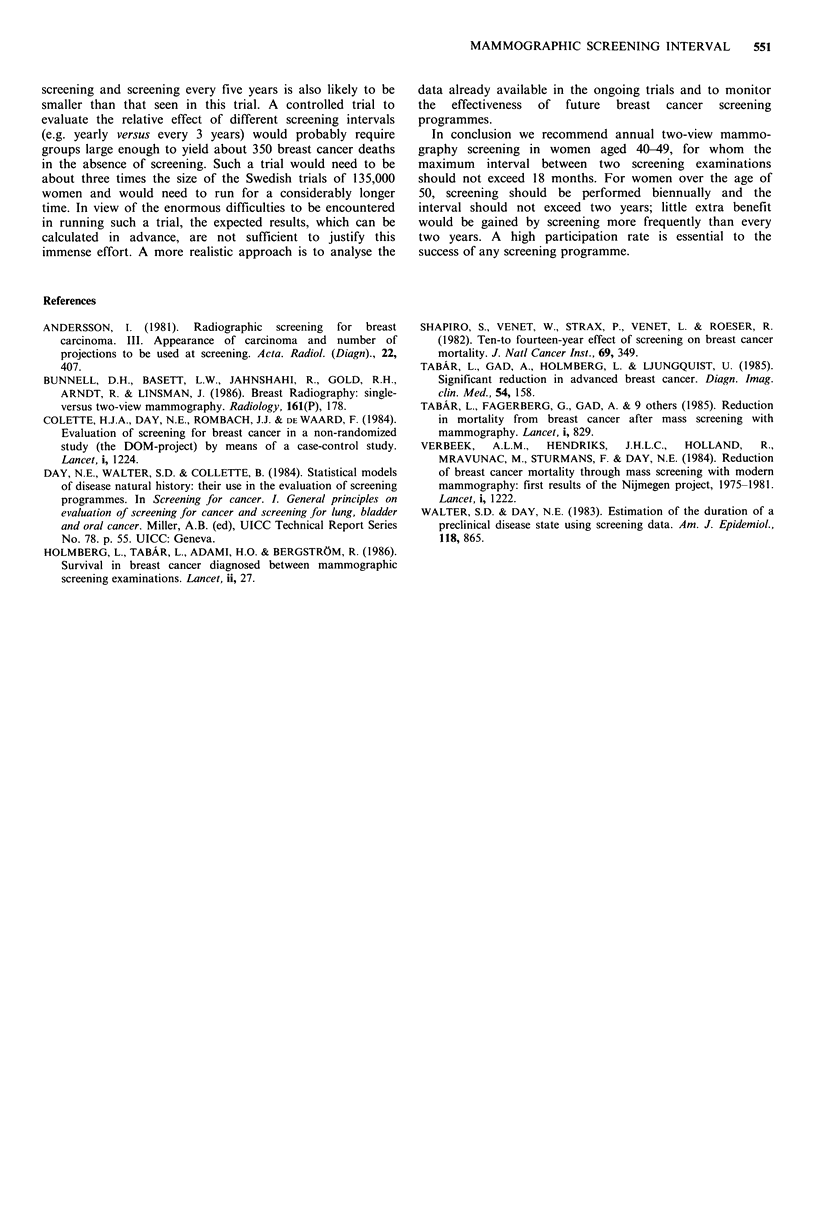# What is the optimum interval between mammographic screening examinations? An analysis based on the latest results of the Swedish two-county breast cancer screening trial.

**DOI:** 10.1038/bjc.1987.112

**Published:** 1987-05

**Authors:** L. Tabár, G. Faberberg, N. E. Day, L. Holmberg

## Abstract

Further results are presented from the Swedish two-county breast cancer screening trial. The reduction in the rate of advanced cancers and of breast cancer mortality in the group allocated to screening when compared to the control group has accelerated with a further year of follow-up. Mortality due to other causes and the rate of other cancers remains similar in the two groups. Attention has been focused on the rate at which cancers start re-emerging among women with negative mammograms. Among women over 50 years of age at entry to the study, relatively few interval cancers are seen in the first two years after a screening test; in the third year the rate rises to nearly 50% of the comparable rate in the control group. Among women aged 40-49 years at entry, by contrast, the rate of interval cancers even in the first post screening year is nearly 40% of that in the controls and in the second year nearly 70%. In older women in the group allocated to screening, much of the breast cancer mortality comes from the refusers and little from the interval cancers; in younger women the picture is reversed. The implications for screening policy, including the interscreening interval are discussed.


*Br. J. Cancer* (1987), **55**, 547–551 © The Macmillan Press Ltd., 1987

### What is the optimum interval between mammographic screening examinations?—An analysis based on the latest results of the Swedish two-county breast cancer screening trial

L. Tabár[1], G. Faberberg[2], N.E. Day[3] & L. Holmberg[4]

[1]*Mammography Department, Central Hospital, Falun, Sweden,* [2]*Department of Radiology, University Hospital, Linköping, Sweden,* [3]*MRC Biostatistics Unit, Cambridge, UK and* [4]*Department of Surgery, Central Hospital, Falun, Sweden.*

**Summary** Further results are presented from the Swedish two-county breast cancer screening trial. The reduction in the rate of advanced cancers and of breast cancer mortality in the group allocated to screening when compared to the control group has accelerated with a further year of follow-up. Mortality due to other causes and the rate of other cancers remains similar in the two groups. Attention has been focused on the rate at which cancers start re-emerging among women with negative mammograms. Among women over 50 years of age at entry to the study, relatively few interval cancers are seen in the first two years after a screening test; in the third year the rate rises to nearly 50% of the comparable rate in the control group. Among women aged 40–49 years at entry, by contrast, the rate of interval cancers even in the first post screening year is nearly 40% of that in the controls and in the second year nearly 70%. In older women in the group allocated to screening, much of the breast cancer mortality comes from the refusers and little from the interval cancers; in younger women the picture is reversed. The implications for screening policy, including the interscreening interval are discussed.

The potential of mass screening to reduce mortality from breast cancer has been clearly demonstrated by the HIP study from New York (Shapiro *et al.*, 1982), the Swedish two-county study (Tabár *et al.*, 1985) and by the studies from the Netherlands (Colette *et al.*, 1984; Verbeek *et al.*, 1984). The qeustions that now arise concern the implementation of breast cancer screening, in particular who should be screened and how often. The two-county Swedish study was designed with a longer inter-screening interval than the HIP or Dutch studies and thus is an invaluable source of information concerning these questions. This longer interval gives the opportunity to analyze the incidence of interval cancers in the second and third years after screening. It is of special interest to examine how the incidence of these interval cancers increases as time elapses since the last screening test (Day *et al.*, 1984; Walter *et al.*, 1983), especially because interval cancers, i.e. breast carcinomas diagnosed between screening examinations, appear to have a similar prognosis to cancers diagnosed in an unscreened population (Shapiro *et al.*, 1982; Holmberg *et al.*, 1986). As the interval cancer incidence approaches to that of the control population, the effect of screening disappears. Thus, a necessary condition for effective screening is that the total incidence of interval cancers is kept relatively low. Our most recent results have given us the opportunity to examine the incidence of interval cancers after the first and second rounds, in each age group. Our purpose is to study the effect of interval length on the efficacy of screening.

### Subjects and methods

The design of the study has been described in detail previously (Tabár *et al.*, 1985). In brief, a total of 162,981 women aged 40 or more were randomly assigned either to be offered or not offered regular single-view mammography at specialised screening centres. The randomisation was performed on a population-block basis consisting of small administrative units (parishes, municipalities). Women over 74 years of age at randomisation had a poor record of attendance and are not considered further in this report. Women aged 70–74 were invited to the first two screening rounds only. Women under 70 at entry have been invited for screening three or four times. Between the first and second screenings the average interval was 33 months for women 50 years of age and older and 24 months for women under 50 years of age. Only women aged 40–69 at entry were invited to the third screening round, for which the average interval since the second screening round was 24 months in all age groups. Overall compliance in the three screening rounds was 89.2%, 83.3% and 84.0% respectively. The average length of follow-up from randomisation to December 31, 1985 is 7.5 years in Kopparberg and 6.5 years in Östergötland. The comparability of the study and control groups was assessed in terms of all causes of death other than from breast cancer up to December 31, 1985 and in terms of deaths from all malignant diseases excluding breast cancer. Both measures are as close as could be expected in the study and control groups.

Screening of the control population started after the completion of the third round of screening in those aged 50 years or more at entry and after the fourth screening round in those under 50 at entry. Breast cancers were treated according to stage at diagnosis and independently of mode of detection. In this report, age refers to age at entry to the study, unless specifically stated otherwise.

### Results

The breast cancer rates in the study and control from randomisation to December 31, 1985 are shown in Table I. Figure 1 demonstrates the evolution of the cumulative rates of advanced cancer in the study and control groups during the progress of the study, for women aged 40–74 at entry. The significance of the difference appears in Table II. Table III gives the mortality from breast cancer in the study and control groups. The cumulative mortality rates in the two groups by year since entry into the study are displayed in Figure 2.

To examine the effect of screening on breast cancer mortality in greater detail we considered the screening status of women in the study group who died from breast cancer (Table IV). Two features of this table are interesting. First, the proportion of deaths found among women who refused screening increases rapidly with age, reaching 50% in the 70–74 age group, and second, the proportion of deaths found among women with interval carcinomas decreases rapidly

Correspondence: L. Tabár
Received 7 October 1986; and in revised form, 5 January 1987.

**Table I** Frequency of breast cancer cases per 1,000 women aged 40–74 at entry diagnosed between the date of randomisation and 31 December 1985

| *The two counties combined* | *Total invasive* | *Stage I* | *Stage II+* | *<20 mm pNX*[a] | *Axillary nodes positive and/or disseminated* | *Ductal in situ* |
|---|---|---|---|---|---|---|
| Study group | 15.3 | 8.9 | 5.9 | 0.6 | 4.1 | 1.4 |
| Control group | 13.1 | 4.8 | 8.0 | 0.3 | 5.0 | 0.4 |

[a]pNX = Invasive cancer, size ≦20 mm, axillary nodes not examined histologically.

**Table II** Comparison between the study and control groups of Stage II or more advanced breast cancers at diagnosis[a]

| | *Kopparberg county* | | *Östergötland county* | |
|---|---|---|---|---|
| | *Stage II+* | *Population* | *Stage II+* | *Population* |
| Study group | 245 | 39,051 | 215 | 39,034 |
| Control group | 174 | 18,846 | 279 | 37,936 |

$\chi_1^2 = 25.0$ ($P < 0.001$), relative risk = 0.72, 95% confidence interval (0.63, 0.81).
[a]Women aged 40–74 at entry.

**Table III** Deaths from breast cancer in the study and control populations[a]

| | *Kopparberg county* | | | *Östergötland county* | | |
|---|---|---|---|---|---|---|
| | *Deaths* | *Populations* | *RR* | *Deaths* | *Population* | *RR* |
| Study group | 71 | 39,051 | 0.66 | 53 | 39,034 | 0.77 |
| Control group | 52 | 18,846 | | 67 | 37,936 | |

Combined $\chi_1^2$ on 1 degree of freedom = 6.9 ($P < 0.01$). Combined estimate of relative risk = 0.71, 95% confidence interval (0.55, 0.91).
[a]Women aged 40–74 at entry.

**Figure 1** Cumulative rates of Stage II and more advanced breast carcinomas per $10^4$ women in the study and control groups, the two counties combined. Women aged 40–74 at entry, +——+ Control; *——* Study.

**Figure 2** Cumulative mortality rates per $10^5$ women by time since randomization in the study and control groups, two counties combined, women aged 40–74 at entry, O——O Study; + — — + Control.

with age. We found that over 50% of deaths from breast cancer in the 40–49 year age group were from these interval carcinomas. Among women dying from breast cancer who attended screening, i.e. diagnosed either at screening or in the interval between two screenings, the proportion arising as interval carcinomas decreases significantly with age ($\chi_1^2 = 4.8$, testing for trend) even though the interval was shorter in younger women. In each age group roughly one-third of the breast cancer deaths were observed in the group diagnosed at screening.

To investigate the ability of mammography to detect early cancers among women of different ages, the incidence of

**Table IV** Deaths from breast cancer in the study group by screening status at the time of diagnosis in the two counties combined

| | *Deaths from breast cancer among women* | | | | |
|---|---|---|---|---|---|
| *Age group* | *Diagnosed in the period between randomisation and invitation to screening* | *Diagnosed at screening* | *Diagnosed in the interval between screenings* | *Diagnosed among non-responders* | *Total* |
| 40–49 | — | 8 | 13 | 2 | 23 |
| 50–59 | 3 | 13 | 8 | 9 | 33 |
| 60–69 | 1 | 17 | 9 | 14 | 41 |
| 70–74 | 1 | 9 | 3 | 14 | 27 |
| All ages | 5 | 47 | 33 | 39 | 124 |

interval cancers in the years succeeding the first and second screening rounds was examined. In each year succeeding the screening test, the number of breast cancer cases that would have been expected to occur if no screening had been performed can be calculated from the incidence in the control group. The difference between this number and the number actually observed gives an estimate of the number of cases picked up at previous screening, which would otherwise have surfaced during the year in question. Table V gives the number of cases observed and the number of expected if no screening had been performed, in the first, second and third years after the first and second screening tests, by decade of age at randomisation. The ratio of these two numbers gives the proportional incidence of interval carcinomas. The results are similar after the first and second screening test and have been pooled in the final column. This column gives the estimated proportion of cases detected at the previous screening, which would have surfaced in each of these yearly intervals. Figure 3 presents graphically the data of Table V giving the incidence of interval cancers as a proportion of the incidence that would be expected in the absence of screening based on the breast cancer incidence in the control group. The contrast between the 40–49 year age group and the older women is striking; in the younger women, the screening test used in this trial picked up about 60% of the breast cancers that would otherwise have surfaced in the succeeding 12 months, and 30% of the breast cancers that would have surfaced in the second year. The corresponding figures for the older women are about 85% and 70%, and even in the third year after screening the test detected 55% of the cases that would have arisen. Little information is available for the third year among the younger women, since screening was scheduled every two years. This difference between the two age groups is highly significant ($\chi^2 = 14.2$; $P < 0.001$). Of the interval cancers in Table V. 58% were

**Figure 3** Breast cancer incidence among screened women in the interscreening interval, in age group 40–49 (a) and 50–69 (b) as a percentage of breast cancer incidence in the control group.

Stage II or worse, a figure close to the percentage of 61% in the control group, as calculated from Table I. Table VI shows the proportion of advanced cancers in each ten year age group among interval cases and those diagnosed at repeat screening, and for comparison, the controls. It should be noted that the interval cancer rates may be underestimated, as the knowledge that a repeat screening appointment is imminent may be disincentive to self-

**Table V** Incidence of interval cancers after the first and second screening tests, relative to the incidence in the control group, by age, the two counties combined

| *Age group* | *Interval after screening (in months)* | *1st interval No. of cases* | | *2nd interval No. of cases* | | *Estimated proportion (%) of cancers detected at previous screen (both intervals combined)* |
|---|---|---|---|---|---|---|
| | | *Observed* | *Expected* | *Observed* | *Expected* | |
| 40–49 | 0–11 | 8 | 20.5 | 7 | 19.2 | 62 |
| | 12–23 | 8 | 16.9 | 15 | 17.0 | 32 |
| | 24–29 | 3 | 5.0 | — | — | — |
| 50–59 | 0–11 | 3 | 40.5 | 6 | 37.7 | 88 |
| | 12–23 | 12 | 40.5 | 10 | 33.1 | 70 |
| | 24–29 | 12 | 26.7 | 4 | 8.2 | 54 |
| 60–69 | 0–11 | 10 | 51.0 | 4 | 44.4 | 85 |
| | 12–23 | 15 | 51.0 | 11 | 40.9 | 72 |
| | 24–29 | 19 | 39.1 | 6 | 16.0 | 55 |

**Table VI** Proportion of Stage II or more advanced cancers among interval cancer cases and among those diagnosed at repeat screening, by age

| | *Proportion of advanced carcinomas* (%) | | |
|---|---|---|---|
| *Age at randomisation* | *Interval cancers* | *Diagnosed at repeat screenings* (*2nd and 3rd combined*) | *Control group* |
| 40–49 | 58.5 | 37.9 | 63.6 |
| 50–59 | 55.3 | 25.0 | 58.7 |
| 60–69 | 58.5 | 16.9 | 58.4 |
| Total | 57.5 | 23.8 | 59.2 |

detection and seeking medical care in the interim. That is, some women may consciously or unconsciously postpone diagnosis until the next screening appointment. To complement Table V and Table VI, Table VII enumerates the breast cancer deaths among women who have been screened. An interesting finding is that deaths from breast cancer among interval cases diagnosed within one year after screening occurred only in the age group 40–49.

### Discussion

Completion of the third round of screening and the average seven year follow up of the control and study populations has enabled us to study the effect of interval length on screening efficacy. There are two major factors which determine the choice of the interscreening interval. These are the proportion of cancers emerging as interval cancers and the prognosis of cancers detected at the repeat screening test. Particular emphasis is given in this report to the former.

Both in this study and in the HIP study the survival of interval cancers has been similar to the survival of cancers in the control group. The reduction of breast cancer mortality should therefore be greater, the lower the proportion of the interval cancers. The results of this study provide a quantitative criterion for comparing different screening frequencies. Although this criterion is not explicitly in terms of reduced breast cancer mortality, it is in terms of a measure directly related to it. This measure, the *proportional incidence of interval cancers*, should give an early assessment of the effectiveness of the screening programme, which depends on both the frequency of screening and the sensitivity of the screening test. The proportion of cancers that appear as interval cancers with different screening intervals is shown in Table V and Figure 3. Among women under age 50 the rate of interval cancers returns to the rate in the control group markedly more rapidly than in older women; in these younger women much of the effect of single-view mammography screening has disappeared in the second year after the screening examination. In the 50–69 age group, the incidence of interval cancers in the third year after screening is nearly half that in the control group of the same age. The information about the *prognosis of cases diagnosed at repeat screening* is not yet complete from this study. There are good indications, however, that the breast cancer mortality in this group will be closely related to the proportion of advanced carcinomas (see Table VI). In these particular cases, screening has not been successful in preventing tumour growth to a potentially fatal stage. The primary reason is not poor sensitivity in the age groups 50–69; the low interval cancer rates during the first two years reflect the high sensitivity of mammography screening in this age group. Stage II and more advanced breast carcinomas accounted for 38% of the cancers detected at repeat screening in age group 40–49; 25% in age group 50–59 and 17% in age group 60–69. Adding these cases to the interval cancer cases, raises the number of carcinomas unaffected by screening to an unacceptably high level during the second year after screening in the age group 40–49 and during the third year after screening in the 50–69 year age group.

Analysis of the screening history of women dying from breast cancer reveals an age-related phenomenon (Table VII). Among women over 70, compliance was low and the effectiveness of the screening programme was correspondingly reduced, more than half of the deaths from breast cancer were observed in those who refused screening. Also, in the 60–69 age group, 34% of the breast cancer deaths occurred among the refusers. Thus, among women 50 and over, low compliance is a major factor impairing the effectiveness of mammography screening. If repeat screening is delayed in more than two years, the increasing rate of both interval cancers and of advanced cancers detected at repeat screening is an additional factor jeopardising the screening effect. By comparison, the sensitivity of screening women 40–49 with single-view mammography every two years is low, because this screening test permits the reappearance of too many interval cancers. These cancers contributed more than half of the total breast cancer deaths in this age group. The sensitivity of the screening examination should be increased; this can be done by using two-view mammography (Andersson *et al.*, 1981; Bunnell *et al.*, 1986). In addition to the low sensitivity, the long interval time (2 years) allowed the occurrence of too many advanced cancers at repeat screening.

It is clear from these results that for women aged 40 to 49 years single-view mammography every two years is not a sufficiently effective screening policy. What is needed is a more sensitive test, more frequently applied; annual two-view mammography would probably provide substantial improvement. For women over the age of 50, there would seem to be little scope for much improvement by screening more frequently than every two years. Less frequent screening will lead to an appreciable increase in the frequency of interval cancers and also in the frequency of Stage II or more advanced tumours. It is tempting to consider randomised trials of the relative effectiveness of different screening intervals for women over 50. The logistics of such trials needs careful evaluation, however. In the Swedish trial, the results to date on mortality reflect mainly the effect of the initial round of screening. No effect was seen for three to four years and significance emerged after some seven years. To evaluate the effect of screening rounds after the first will clearly take considerably longer. The expected difference in breast cancer between, say, yearly

**Table VII** Screening history of women dying from breast cancer

| *Age group* | *Diagnosed at first screening* | *Interval cancers, months between diagnosis and last negative screening* 0–11 | 12–23 | 24–35 | 36+ | *Diagnosed second or third screening* |
|---|---|---|---|---|---|---|
| 40–49 | 5 | 5 | 5 | 3 | | 3 |
| 50–59 | 9 | | 4 | 3 | 1 | 4 |
| 60–69 | 15 | | 3 | 6 | | 2 |
| Total | 29 | 5 | 12 | 12 | 1 | 9 |

screening and screening every five years is also likely to be smaller than that seen in this trial. A controlled trial to evaluate the relative effect of different screening intervals (e.g. yearly *versus* every 3 years) would probably require groups large enough to yield about 350 breast cancer deaths in the absence of screening. Such a trial would need to be about three times the size of the Swedish trials of 135,000 women and would need to run for a considerably longer time. In view of the enormous difficulties to be encountered in running such a trial, the expected results, which can be calculated in advance, are not sufficient to justify this immense effort. A more realistic approach is to analyse the data already available in the ongoing trials and to monitor the effectiveness of future breast cancer screening programmes.

In conclusion we recommend annual two-view mammography screening in women aged 40–49, for whom the maximum interval between two screening examinations should not exceed 18 months. For women over the age of 50, screening should be performed biennually and the interval should not exceed two years; little extra benefit would be gained by screening more frequently than every two years. A high participation rate is essential to the success of any screening programme.

### References

ANDERSSON, I. (1981). Radiographic screening for breast carcinoma. III. Appearance of carcinoma and number of projections to be used at screening. *Acta. Radiol.* (*Diagn*)., **22,** 407.

BUNNELL, D.H., BASETT, L.W., JAHNSHAHI, R., GOLD, R.H., ARNDT, R. & LINSMAN, J. (1986). Breast Radiography: single- versus two-view mammography. *Radiology,* **161**(P), 178.

COLETTE, H.J.A., DAY, N.E., ROMBACH, J.J. & DE WAARD, F. (1984). Evaluation of screening for breast cancer in a non-randomized study (the DOM-project) by means of a case-control study. *Lancet,* **i,** 1224.

DAY, N.E., WALTER, S.D. & COLLETTE, B. (1984). Statistical models of disease natural history: their use in the evaluation of screening programmes. In *Screening for cancer. I. General principles on evaluation of screening for cancer and screening for lung, bladder and oral cancer.* Miller, A.B. (ed), UICC Technical Report Series No. 78. p. 55. UICC: Geneva.

HOLMBERG, L., TABÁR, L., ADAMI, H.O. & BERGSTRÖM, R. (1986). Survival in breast cancer diagnosed between mammographic screening examinations. *Lancet,* **ii,** 27.

SHAPIRO, S., VENET, W., STRAX, P., VENET, L. & ROESER, R. (1982). Ten-to fourteen-year effect of screening on breast cancer mortality. *J. Natl Cancer Inst.,* **69,** 349.

TABÁR, L., GAD, A., HOLMBERG, L. & LJUNGQUIST, U. (1985). Significant reduction in advanced breast cancer. *Diagn. Imag. clin. Med.,* **54,** 158.

TABÁR, L., FAGERBERG, G., GAD, A. & 9 others (1985). Reduction in mortality from breast cancer after mass screening with mammography. *Lancet,* **i,** 829.

VERBEEK, A.L.M., HENDRIKS, J.H.L.C., HOLLAND, R., MRAVUNAC, M., STURMANS, F. & DAY, N.E. (1984). Reduction of breast cancer mortality through mass screening with modern mammography: first results of the Nijmegen project, 1975–1981. *Lancet,* **i,** 1222.

WALTER, S.D. & DAY, N.E. (1983). Estimation of the duration of a preclinical disease state using screening data. *Am. J. Epidemiol.,* **118,** 865.